# Brain AVMs-Related microRNAs: Machine Learning Algorithm for Expression Profiles of Target Genes

**DOI:** 10.3390/brainsci12121628

**Published:** 2022-11-28

**Authors:** Alice Giotta Lucifero, Sabino Luzzi

**Affiliations:** 1 Neurosurgery Unit, Department of Clinical-Surgical, Diagnostic and Pediatric Sciences, University of Pavia, 27100 Pavia, Italy; 2 Neurosurgery Unit, Department of Surgical Sciences, Fondazione IRCCS Policlinico San Matteo, 27100 Pavia, Italy

**Keywords:** artificial intelligence, brain arteriovenous malformations (AVM), hemorrhagic stroke, machine learning, microRNA, non-coding RNA, VEGF

## Abstract

Introduction: microRNAs (miRNAs) are a class of non-coding RNAs playing a myriad of important roles in regulating gene expression. Of note, recent work demonstrated a critical role of miRNAs in the genesis and progression of brain arteriovenous malformations (bAVMs). Accordingly, here we examine miRNA signatures related to bAVMs and associated gene expression. In so doing we expound on the potential prognostic, diagnostic, and therapeutic significance of miRNAs in the clinical management of bAVMs. Methods: A PRISMA-based literature review was performed using PubMed/Medline database with the following search terms: “brain arteriovenous malformations”, “cerebral arteriovenous malformations”, “microRNA”, and “miRNA”. All preclinical and clinical studies written in English, regardless of date, were selected. For our bioinformatic analyses, miRWalk and miRTarBase machine learning algorithms were employed; the Kyoto Encyclopedia of Genes and Genomes (KEGG) database was quired for associated pathways/functions. Results: four studies were ultimately included in the final analyses. Sequencing data consistently revealed the decreased expression of miR-18a in bAVM-endothelial cells, resulting in increased levels of vascular endodermal growth factor (VEGF), Id-1, matrix metalloproteinase, and growth signals. Our analyses also suggest that the downregulation of miR-137 and miR-195* within vascular smooth muscle cells (VSMCs) may foster the activation of inflammation, aberrant angiogenesis, and phenotypic switching. In the peripheral blood, the overexpression of miR-7-5p, miR-629-5p, miR-199a-5p, miR-200b-3p, and let-7b-5p may contribute to endothelial proliferation and nidus development. The machine learning algorithms employed confirmed associations between miRNA-related target networks, vascular rearrangement, and bAVM progression. Conclusion: miRNAs expression appears to be critical in managing bAVMs’ post-transcriptional signals. Targets of microRNAs regulate canonical vascular proliferation and reshaping. Although additional scientific evidence is needed, the identification of bAVM miRNA signatures may facilitate the development of novel prognostic/diagnostic tools and molecular therapies for bAVMs.

## 1. Introduction

Brain arteriovenous malformations (bAVMs) are rare vascular abnormalities with an incidence of 1.34/100,000 patient-years and a prevalence ranging from 10 to 18 in 100,000 adults [1,2,3,4]. They consist of an intraparenchymal tangle of small dysplastic vessels, called a nidus, which directly connects the arterial and venous systems without a traversing capillary bed. Intranidal vessels undergo persistently elevated hemodynamic forces which create a high-flow shunt between feeding arteries and draining veins [5,6]. As such, bAVMs account for ~1–2% of hemorrhagic strokes; the annual risk of rupture is ~2–4% per year, with rates that essentially double after an index bleed [1,2,3,7,8,9]. Neurological sequelae have been described in 20–30% of patients; the mortality rate approaches 10% and depends on a litany of additional factors, (e.g., grading score, patient age, ethnicity, and/or previous hemorrhage) [2,5,7,10,11,12].

Despite vascular malformations having been defined for years as congenital/static lesions, advanced genomic techniques have come to identify solitary mutations and single-nucleotide polymorphisms that drive sporadic bAVMs [13,14,15,16,17,18].

Genetic perturbations when combined with hemodynamic stimuli, may therefore trigger biomolecular mechanisms underlying the pathobiology/pathogenesis of bAVMs [5,19,20]. Given this, the importance of modern sequencing analyses is evident as one looks to elucidate the gene-based regulatory networks involved in bAVMs growth and progression. 

microRNAs (miRNAs) are a class of non-coding RNAs that play important roles in regulating gene expression and have been shown capable of influencing endothelial homeostasis, inflammation, and vascular architecture remodeling [21,22]. The dysregulation of miRNAs’ expression profiles has the potential to alter endothelial-smooth muscle cell interactions, resulting in aberrant angiogenesis, tube formation, and bAVMs reorganization [21,23,24]. Understanding such network regulation offers opportunities to design novel diagnostics and therapeutics capable of interceding in the pathogenesis of bAVMs. 

The present article aims to provide a descriptive synopsis of the miRNA signatures found in bAVMs/the associated microenvironment with an emphasis on miRNA-related target genes in cell proliferation, vascular rearrangement, and/or inflammation. Through artificial intelligence and machine learning algorithms, bioinformatics analyses were carried out to examine miRNA expression profiles and related downstream pathways as potential causative factors, novel diagnostic and prognostic biomarkers, and possible therapeutic targets capable of assisting in the management of these complex vascular malformations.

## 2. Materials and Methods

### 2.1. Data Sources and Inclusion Criteria 

A literature review was performed in accordance with the Preferred Reporting Items for Systematic Reviews and Meta-Analyses (PRISMA) guidelines [25]. We quired the PubMed/Medline (https://pubmed.ncbi.nlm.nih.gov (accessed on 16 July 2022)) electronic database using combinations of the following search terms and idioms: “brain arteriovenous malformations”, “cerebral arteriovenous malformations”, “bAVM”, “microRNA”, “non-coding RNA”, “miRNA”, and “miR”. All preclinical and clinical studies written in English, regardless of date, were selected. We included clinical studies on humans and surgical specimens, as well as experimental articles related to pertinent animal models and relevant in vitro studies. Review articles, editorials, and case reports were excluded. Results were further sorted based on their relevance as inferred from a review of titles/abstracts. Our data extraction protocol recorded the following information: authors’ names, year/country of publication, miRNA status, and levels of potential mRNA target expression. 

### 2.2. Bioinformatic Analyses

We performed a series of bioinformatic analyses to further define the potential functional roles of miRNA-related target networks and molecular pathways in bAVMs. Potential mRNA-related targets were obtained through the miRWalk machine-learning algorithm (http://mirwalk.umm.uni-heidelberg.de (accessed on 16 July 2022)) [26]. 

Through this database, the target mining function allows the prediction of the miRNA-binding sites. Furthermore, the advanced profiling analysis of post-transcriptional signals builds maps of regulatory networks linked to the differentially expressed miRNAs [26].

The regions of interest selected were the 5′-UTR, CDS, and 3′-UTR, while the predicted targets were ranked according to preset miRabel’s score (http://bioinfo.univ-rouen.fr/mirabel/; accessed on 30 August 2022). 

The expression profiles of mRNA targets of microRNAs were further investigated via the miRTarBase (http://mirtarbase.cuhk.edu.cn (accessed on 16 July 2022)). miRNAs, putative targets, level of expression in human tissue, and associated pathologies were derived [27]. 

Functional enrichment analysis of target genes was performed using the Kyoto Encyclopedia of Genes and Genomes (KEGG) database [28].

## 3. Results

### 3.1. Literature Review and Data Extraction

**Figure 1:**
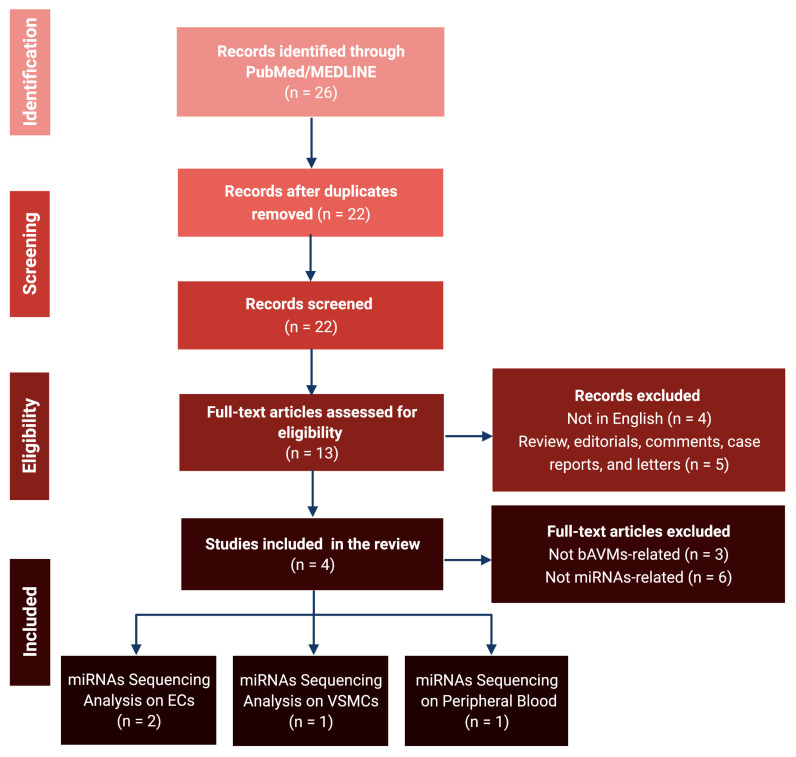
PRISMA flowchart for the review. bAVM: Brain Arteriovenous Malformation; ECs: Endothelial Cells; miRNA: microRNA; VSMCs: Vascular Smooth Muscle Cells.

Our literature search returned 26 records; after the removal of duplicates and the application of our inclusion criteria, four items were deemed eligible for inclusion within the review. The PRISMA flow chart outlining the search strategy is presented in Figure 1. 

Eight miRNAs were detected via genetic investigation(s) of surgically resected tissue and in vitro samples of bAVM-derived endothelial cells (bAVM-ECs), vascular smooth muscle cells (VSMCs), and peripheral blood. Human bAVMs samples were obtained after microsurgical resection. The onset, sporadic nature, or inherited genetic disease was undefined, except for Chen’s series. This latter included only patients harboring unruptured bAVMs [29,30,31,32].

Table 1 summarizes the genetics and expression profiles of the miRNAs identified.

**Figure 2:**
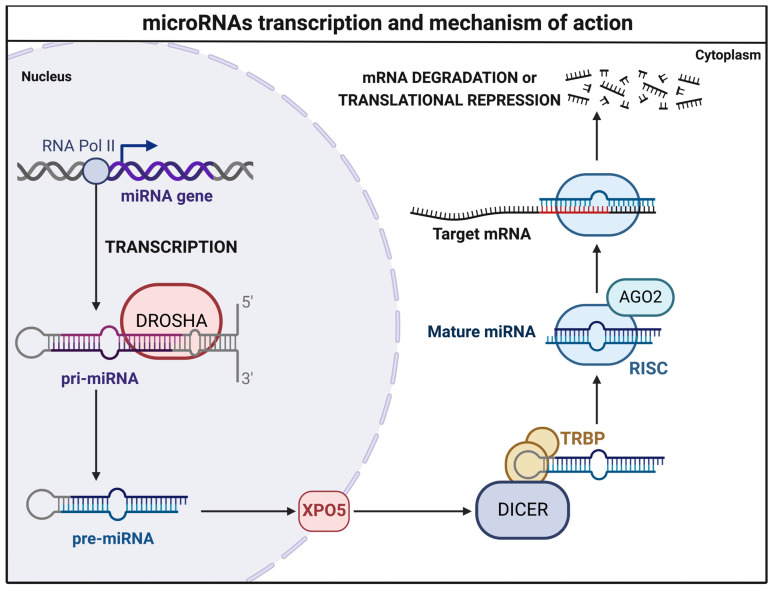
miRNA transcription and mechanism of action. RNA polymerase II (RNA Pol II) drives the transcription of primary microRNA (pri-miRNA) from the *miRNA* gene. Pri-miRNA is then processed by the Double-Stranded RNA-Specific Endoribonuclease (DROSHA), an RNase III enzyme, into 60- to 110-nucleotides pre-miRNA within the nucleus. The pre-miRNA is transferred to the cytoplasm by the Exportin-5 (XPO5). pre-miRNA is then cleaved by the RNase DICER, combined with the transactivation response element RNA-binding protein (TRBP), an RNA-binding cofactor, into a mature miRNA duplex. The miRNA is loaded on AGO2, an endonucleolytic component of the RISC complex (RNA-induced silencing complex), and split. The miRNA antisense strand binds the target messenger RNA (mRNA), assembling a double-stranded helix. If there is a total complementarity, mRNA undergoes endonucleolytic degradation. Contrastingly, in the case of partial complementarity, miRNA is translationally repressed.

Figure 2 summarizes the general mode of action of miRNAs, their impact on target mRNA expression, and modulations (Figure 2).

### 3.2. MicroRNA Sequencing Analyses

#### 3.2.1. miR-18a

Robust miRNA sequencing was performed by Ferreira and colleagues in 2014 [29]; they undertook the deep genetic profiling of human bAVM-ECs. ECs own atypical features and intense proliferation pathways are upregulated by the overexpression of endothelial growth factors [22,33]. Critically, miRNA-18a (miR-18a) was found to be downregulated in bAVM-ECs as compared to ECs from the healthy cortex (i.e., a 2.94-fold decrease). The canonical function of miR-18a centers on its regulatory role in angiogenesis; this is accomplished via the inhibition of proangiogenic factors in part via the silencing of the inhibitor of DNA-binding protein 1 (Id-1), a transcriptional repressor of the thrombospondin-1 (TSP-1) [34,35]. 

Accordingly, under-expression of miR-18a and TSP-1 results in uncontrolled expression of Id-1, leading to an increased level of the vascular endothelial growth factors (VEGF)-A and -D and aberrant vascular proliferation [13,29]. In line with this, the miR-18a was shown to modulate TSP-1 transcription with associated reductions in Id-1 and VEGFs expression, thereby normalizing bAVMs’ growth rate [29].

**Figure 3:**
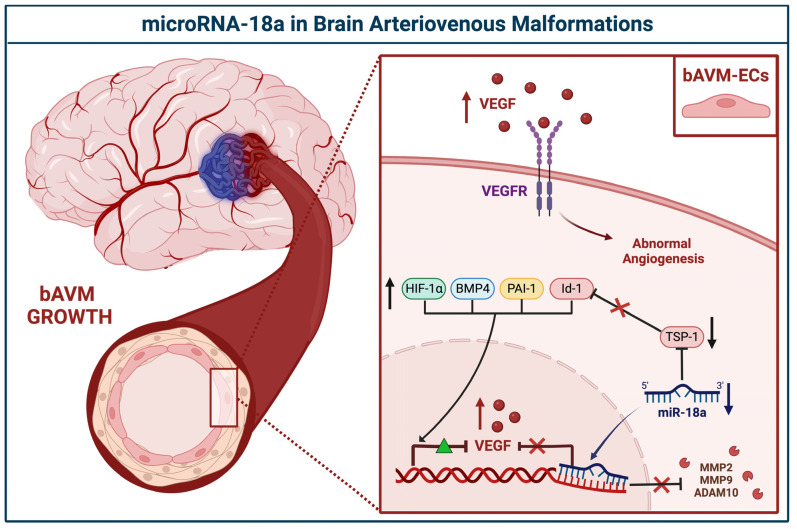
Schematic representation of the microRNA-18a in the pathogenesis of brain AVMs. In the bAVM-derived endothelial cells (bAVM-ECs), the downregulation of miRNA-18a (miR-18a) directly induces an increase in vascular endothelial growth factors (VEGF) transcription, which leads to intense aberrant angiogenesis. The simultaneous repression of thrombospondin-1 (TSP-1) lowers the inhibition of hypoxia-inducible factor 1α (HIF-1α), bone morphogenetic protein 4 (BMP4), plasminogen activator inhibitor-1 (PAI-1), DNA-binding protein 1 (Id-1), matrix metalloproteinases (MMP2 and 9), and ADAM metallopeptidase domain 10 (ADAM10); all involved in vascular proliferation and rearrangement.

In 2020, Marín-Ramos et al. confirmed the downregulation of miR-18a in human surgical specimens of bAVM-ECs [30]. As part of this work, they also sought to use miR-18a as a potential non-invasive therapy to restore the normal bECs phenotype(s) and in so doing revert the bAVMs pathogenesis/progression. miR-18a was administered to murine AVM models; Mgp−/− treated mice underwent computed tomography angiography (CTA) which served to demonstrate the effectiveness of the drug in reducing aberrant neoangiogenesis and AVM development [30]. Protein expression analysis confirmed the involvement of miR-18a in the antiangiogenetic pathways impeding the expression of VEGFs through the inhibition of the plasminogen activator inhibitor-1 (PAI-1), bone morphogenetic protein 4 (BMP4), and hypoxia-inducible factor 1α (HIF-1α) [30,36]. miR-18a demonstrated additional roles in the rearrangement of matrix proteins within the microenvironment bordering bAVM via a reduction in the secretion of matrix metalloproteinases (MMP2 and 9) and ADAM metallopeptidase domain 10 (ADAM10) [30] (Figure 3).

#### 3.2.2. miR-137 and miR-195*

The histopathological hypertrophy of bAVM vascular walls provided the theoretical basis for the genetic analysis of the cross-link between VSMCs, biomolecular growth signals, and inflammation pathways [37]. 

In 2017, Huang et al. explored the miRNA signatures in the bAVM and the effects on the VSMCs phenotype [31]. miR-137 and miR-195* were found to be downregulated, while the related downstream pathways increased. 

Proteomics analysis revealed an upsurge in VEGFs, RAS-related pathways, such as the phosphoinositide 3-kinase (PI3K)/protein kinase D1(PDK1)/protein kinase B (Akt) and rapidly accelerated fibrosarcoma kinases (Raf)/mitogen-activated protein kinase (MAP kinase)/extracellular signal-regulated kinase (ERK), and nuclear factor kappa-light-chain of activated B cells (NFkB), all bonded to aberrant angiogenesis processes [31].

Assuming the hypothetical role of angiogenesis suppression, miR-137 and miR-195* were then transfected to the bAVM-VSMCs. Results displayed the restoration of normal cellular functions and the inhibition of proliferation [31].

**Figure 4:**
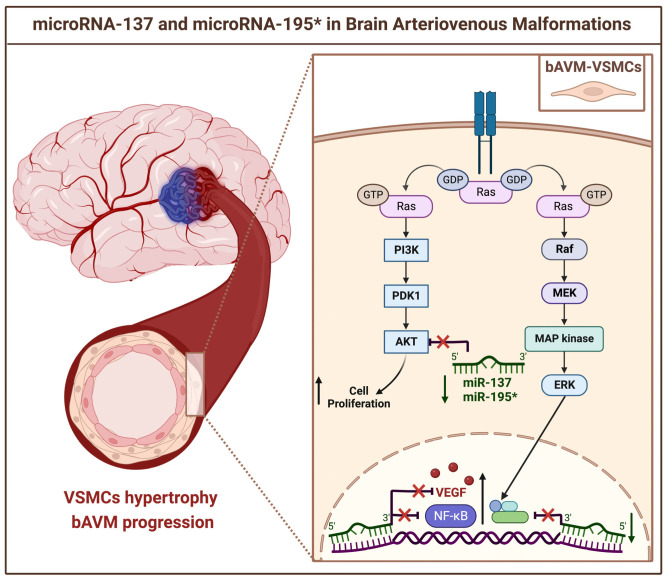
Schematic representation of the microRNA-137 and -195* in the pathogenesis of brain AVMs. The downregulation of miR-137 and miR-195* in the vascular smooth muscle cells (VSMCs) increases the RAS-related pathways, such as the phosphoinositide 3-kinase (PI3K)/protein kinase D1(PDK1)/protein kinase B (Akt) and rapidly accelerated fibrosarcoma kinases (Raf)/mitogen-activated protein kinase (MAP kinase)/extracellular signal-regulated kinase (ERK), all involved in VSMCs phenotype switching and cellular growth. The reduced activity of miR-137 and miR-195* also triggers the transcription of vascular endothelial growth factor (VEGF) and nuclear factor kappa-light-chain of activated B cells (NFkB), all linked to inflammation signals and aberrant angiogenesis. GDP: Guanosine diphosphate; GTP: Guanosine 5′-Triphosphate.

These pieces of evidence suggested the noticeable role of miR-137 and miR-195 in VSMCs phenotypic switching and tube development (Figure 4).

#### 3.2.3. Peripheral Blood miRNAs

In 2018, Chen and colleagues performed a wide miRNA next-generation gene sequencing on peripheral blood samples obtained from three patients harboring bAVMs compared to healthy subjects [32]. In the study group, 246 miRNAs were found to be dysregulated. The top five miRNAs, found at high serum levels, were as follows: miR-7-5p, miR-629-5p, miR-199a-5p, miR-200b-3p, and let-7b-5p. The functional enrichment analysis of potential target networks showed the above-mentioned miRNAs were all implicated in the vascular rearrangement. The increased levels of miRNAs regulate multiple pathways implicated in bAVMs pathogenesis, triggering growth factors, vascular adhesion molecules, and inflammatory mediators. Amid the enriched regulatory networks of aberrant angiogenesis, the VEGF signaling cascade was pivotal in supporting tube formation, bECs proliferation, and migration [32].

**Figure 5:**
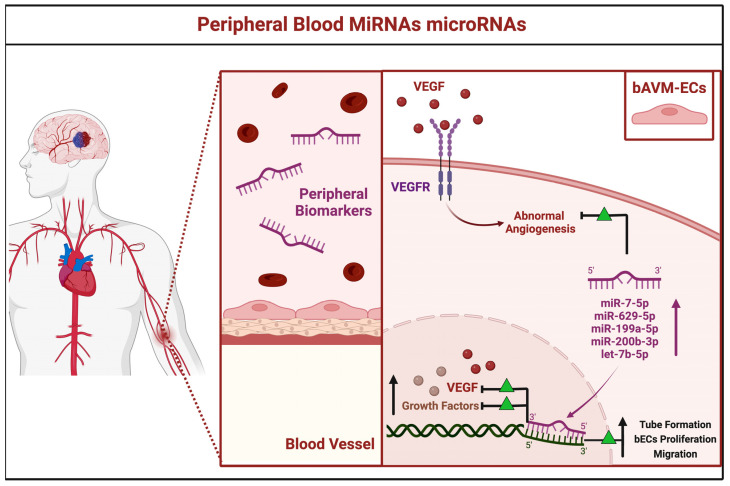
Schematic representation of the peripheral blood microRNAs. The microRNA-7-5p, miR-629-5p, miR-199a-5p, miR-200b-3p, and let-7b-5p are found raised in the serum to be identifiable as peripheral biomarkers. They regulate multiple pathways involved in the vascular re-arrangement, bAVMs growth, and inflammation processes, via the activation of vascular endothelial growth factor (VEGF), vascular endothelial growth factor receptor (VEGFR), and growth factors.

Furthermore, the miR-7-5p, -629-5p, -199a-5p, -200b-3p, and let-7b-5p, identified in the serum, may be interpreted as peripheral biomarkers of diagnosis, grim prognosis, and potential therapeutic targets for bAVMs (Figure 5).

### 3.3. Prediction of miRNA-Related Targets

**Figure 6:**
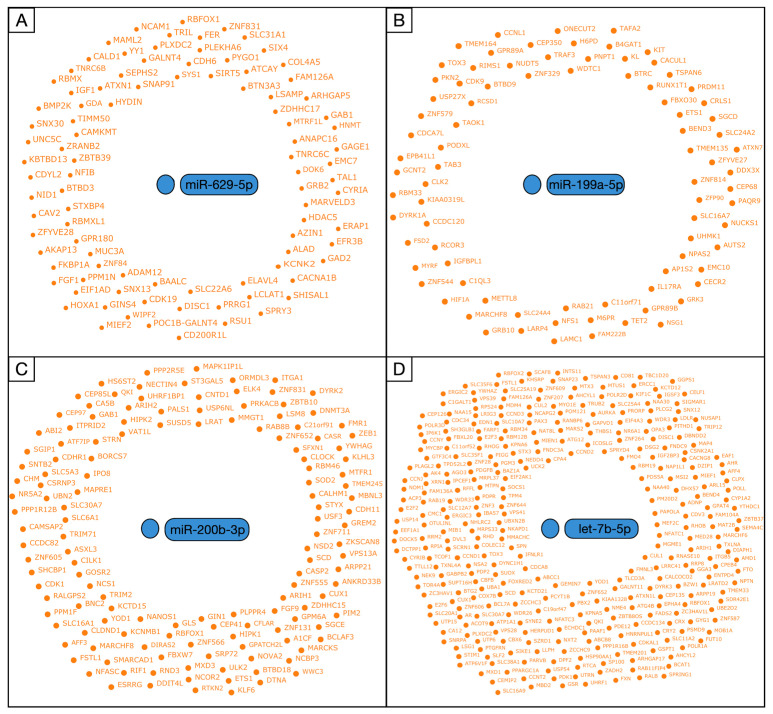
Graphical representation of targets and regulatory networks for the miR-629-5p (**A**), miR-199a-5p (**B**), miR-200b-3p (**C**), and let-7b-5p (**D**).

The machine learning allowed us to scan the gene target networks for the miR-629-5p, miR-199a-5p, miR-200b-3p, and let-7b-5p [26] (Figure 6).

**Figure 7:**
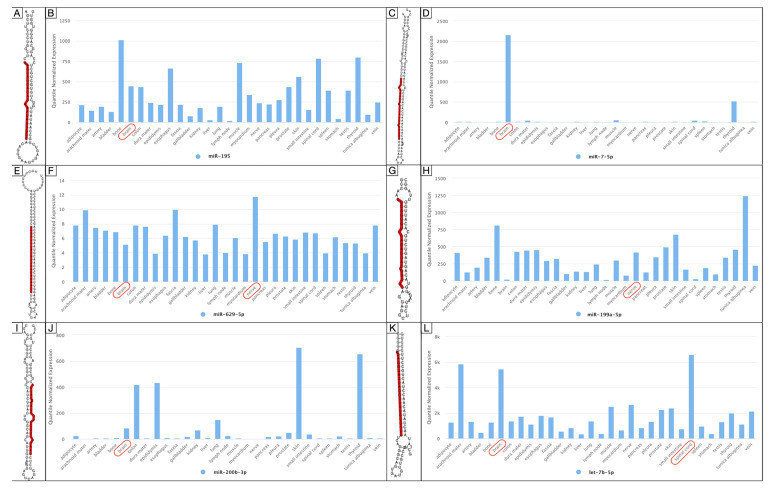
RNA secondary structure and the quantile normalized expression in human organs for miR-195* (**A**,**B**), miR-7-5p (**C**,**D**), miR-629-5p (**E**,**F**), miR-199a-5p (**G**,**H**), miR-200b-3p (**I**,**J**), and let-7b-5p (**K**,**L**). Red circled text shows the nervous system structures involved.

Profiling analysis returned the mature miRNAs, RNA secondary structure, and the quantile normalized expression in human tissue for miR-195*, miR-7-5p, miR-629-5p, miR-199a-5p, miR-200b-3p, and let-7b-5p. miR-195*, miR-7-5p, miR-629-5p, miR-200b-3p, and let-7b-5p were found predominantly expressed in the brain, while miR-199a-5p and let-7b-5p were in the nerves and spinal cord, respectively [27] (Figure 7).

**Figure 8:**
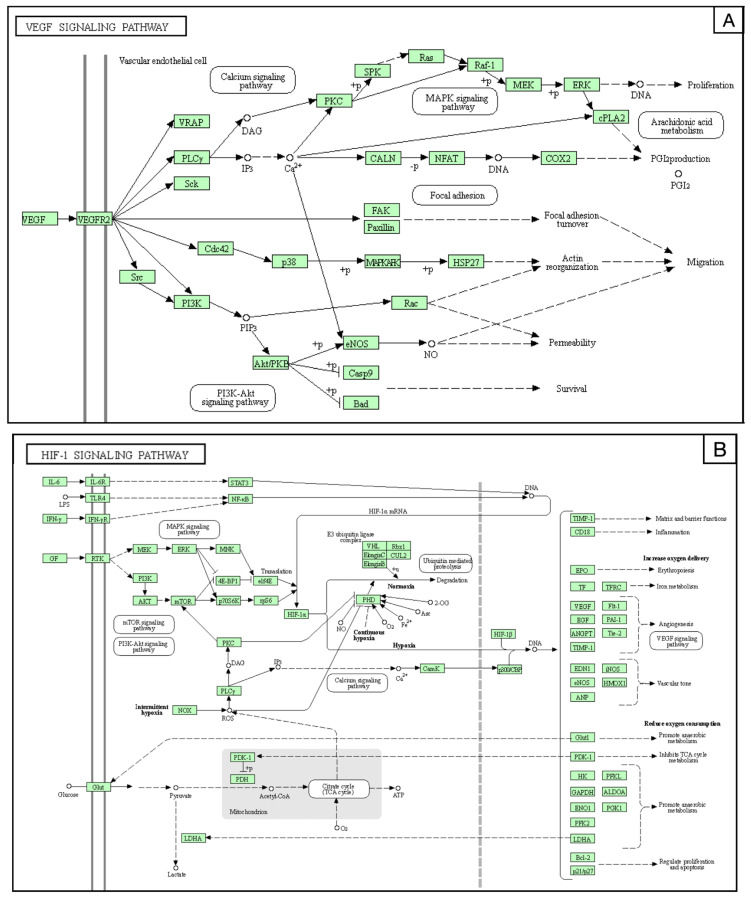
(**A**) VEGF regulated genes and signaling pathways. The vascular endothelial growth factor (VEGF) pathway activates several signaling pathways, which results in the upregulation of genes involved in the survival, proliferation, and migration of endothelial cells and vascular permeability. The binding of VEGF to VEGFR-2 leads to dimerization of the receptor, followed by intracellular activation of the VRAP and PLCγ, linked to the calcium signaling pathway; phosphoinositide 3-kinase (PI3K)/protein kinase D1(PDK1)/protein kinase B (Akt); and rapidly accelerated fibrosarcoma kinases (Raf)/mitogen-activated protein kinase (MAP kinase)/extracellular signal-regulated kinase (ERK) involved in DNA synthesis and cell growth. Activation of PI3K, FAK, and p38 MAPK is implicated in cell migration signaling. (**B**) HIF-1α regulated genes and signaling pathways. Hypoxia-inducible factor 1 (HIF-1) is the master intracellular regulator of oxygen homeostasis. It consists of two subunits: the inducibly-expressed HIF-1α and the constitutively expressed HIF-1β subunit. Under hypoxia, the HIF-1α interacts with coactivators, such as p300/CBP, to modulate the transcription of multiple hypoxia-inducible genes. Among the target genes are the vascular endothelial growth factor (VEGF), epidermal growth factor (EGF), erythropoietin (EPO), endothelial nitric oxide synthase (eNOS), glucose transporter (GLUT), and glyceraldehyde-3-phosphate dehydrogenase (GAPDH), inducing angiogenesis in response to reduced oxygen availability, cell proliferation, vascular tone, erythropoiesis, iron, and anaerobic metabolism, respectively.

Furthermore, the bioinformatic analysis on the KEGG database retrieved a functional enrichment appraisal of miRNA-related intracellular signals involved in the pathogenesis of bAVMs [28]. The main pathway, modulated by the miRNAs, was the VEGF, which regulates the aberrant angiogenesis and remodeling of the nidus (Figure 8A).

miR-18a was proved to downregulate the HIF-1α function. The HIF-1α pathway promotes cell proliferation, metabolism, bECs growth, and vascular tone (Figure 8B). 

**Figure 9:**
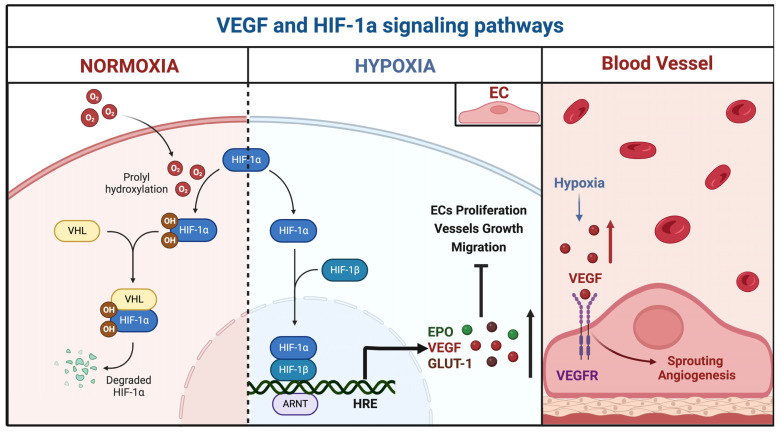
Hypoxia-inducible factor 1 (HIF-1α), in normoxia conditions, undergoes hydroxylation at specific prolyl residues. It is identified by the von Hippel–Lindau tumor-suppressor protein (VHL) and this interaction promotes its rapid ubiquitination and degradation. Under hypoxia, the complex HIF-1α/β, combined with the aryl hydrocarbon receptor nuclear translocator (ARNT) and the transcriptional regulator hypoxia-response element (HRE), induces transcription of erythropoietin (EPO), vascular endothelial growth factor (VEGF), and glucose transporter 1 (GLUT1). These pathways regulate, erythropoiesis, cell metabolism, and migration. Furthermore, the high blood level of VEGF promotes endothelial proliferation and angiogenesis. vascular endothelial growth factor receptor (VEGFR).

Figure 9 reports the interaction between the VEGF and HIF-1 signaling pathways. Hypoxia triggers pathological molecular mechanisms underlying the progression of neurovascular pathologies, such as bAVMs (Figure 9).

**Figure 10:**
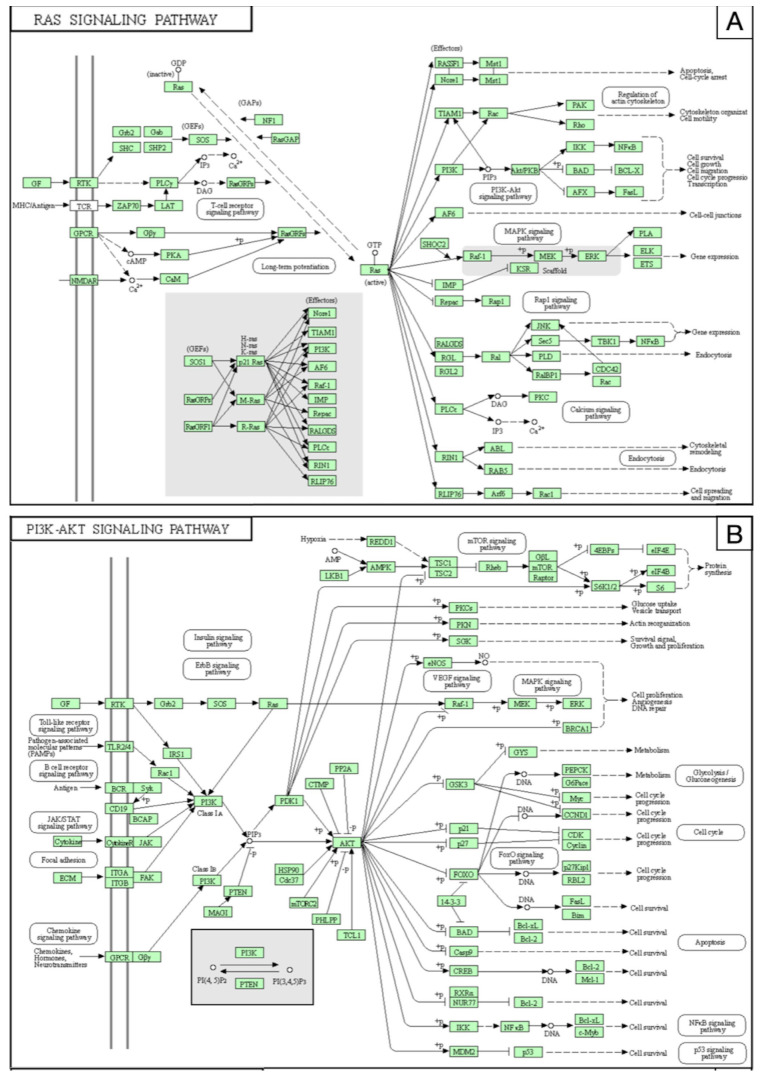

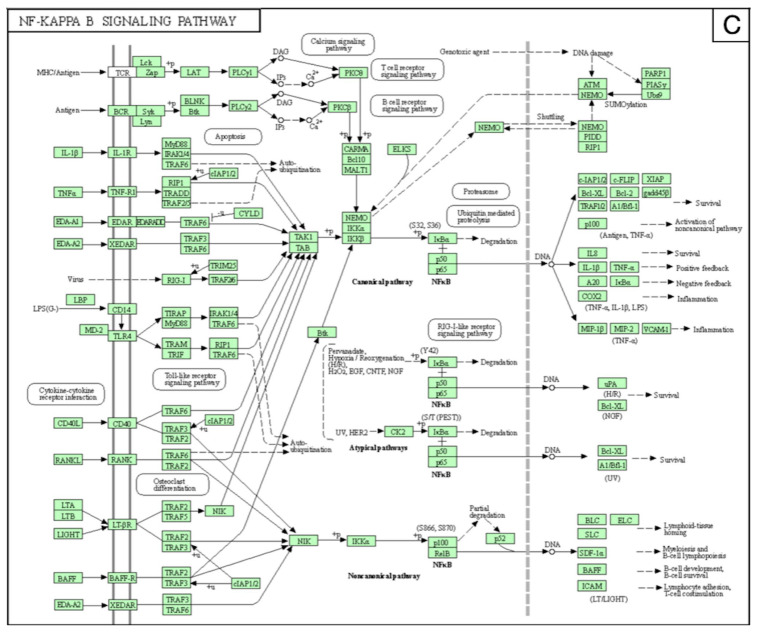
(**A**) The RAS proteins function as molecular switches for signaling pathways regulating cell proliferation, survival, growth, migration, differentiation, or cytoskeletal dynamism. Ras proteins transduce signals from extracellular growth factors by cycling between inactive nucleotide guanosine diphosphate (GDP)-bound and active nucleotide guanosine diphosphate triphosphate (GTP)-bound states. The exchange of GTP for GDP on RAS is regulated by guanine nucleotide exchange factors (GEFs) and GTPase-activating proteins (GAPs). Activated RAS (RAS-GTP) regulates multiple cellular functions through effectors including Raf, phosphatidylinositol 3-kinase (PI3K), Ral guanine nucleotide-dissociation stimulator (RALGDS), calcium signaling pathways, cell endocytosis, migration, and survival. (**B**) The phosphatidylinositol 3′-kinase (PI3K)-Akt signaling pathway is activated by many types of cellular stimuli or toxic insults and regulates fundamental cellular functions such as transcription, translation, proliferation, growth, and survival. The binding of growth factors to their receptor tyrosine kinase (RTK) or G protein-coupled receptors (GPCR) stimulates class Ia and Ib PI3K isoforms, respectively. PI3K catalyzes the production of phosphatidylinositol-3,4,5-triphosphate (PIP3) at the cell membrane. PIP3 in turn serves as a second messenger that helps to activate Akt. Once active, Akt can control key cellular processes by phosphorylating substrates involved in apoptosis, protein synthesis, glucose metabolism, and cell cycle. (**C**) Nuclear factor-kappa B (NFkB) is a transcription factor that regulates mechanisms of immunity, inflammation, and cell survival. The canonical pathway of NFkB is activated by the tumor necrosis factor-alpha (TNF-alpha), interleukin-1 (IL-1), or infections. These signals rely on the IkB kinase (IKK)-mediated IkB-alpha phosphorylation on Ser32 and 36, leading to its degradation. This allows the p50/p65 NF-kB dimer to penetrate the nucleus and activate gene transcription. Atypical IKK-independent pathways rely on the phosphorylation of IkB-alpha on Tyr42 or Ser residues in the IkappaB-alpha PEST domain. The non-canonical pathway is triggered by members of the tumor necrosis factor receptor 1 (TNFR) superfamily, such as lymphotoxin-beta (LT-beta) or B-cell activating factor (BAFF). It involves NIK and IKK-alpha-mediated p100 phosphorylation and processing to p52, resulting in the nuclear translocation of p52/RelB heterodimers, involving in B-cell survival and lymphopoiesis.

miR-137 and miR-195*, expressed within the bAVM-VSMCs, inhibit the RAS pathway and MAPK/ERK (Figure 10A), PI3K/Akt (Figure 10B), and NFkB (Figure 10C) pathways, which control the cell cycle, spreading, survival, gene expression, and activation of the inflammatory cascade.

All the miRNA-target regulatory networks are interconnected and converge in the modulation of pro-inflammatory, proangiogenetic pathways, and gene transduction aimed at dynamically enhancing bAVMs growth.

## 4. Discussion

The present article was aimed at identifying the miRNAs involved in bAVMs pathogenesis, related downstream genetic networks, the potential significance as prognostic biomarkers, and their diagnostic and therapeutic role. 

Current shreds of evidence in the literature report the bAVMs as dynamic and mutable lesions, supported by constant vascular remodeling under the stimulus of blood flow [5,38,39]. They consist of enlarged arterial feeders and draining veins, which shunt in a nidus of tangled vessels. The direct communication of arterial and venous blood pressures, without capillary interposition, explains the high blood flow within the nidus and the mild hemorrhage rate ranging from 2% to 4% per year [40,41].

The exact molecular mechanisms underlying bAVMs growth, pathogenesis, and evolution are still debated. Some congenital hereditary diseases were identified as correlated, for example, hereditary hemorrhagic telangiectasia (HHT), Sturge–Weber disease, and Osler–Weber–Rendu syndrome [17,42,43,44,45]. 

In 2017, Brinjikji and colleagues conducted a systematic review and meta-analysis of the correlation between bAVMs and HHT. They concluded that the bAVMs have a prevalence of approximately 10% in the HHT population, with a greater incidence of HHT1. HHT-related bAVMs are usually symptomatic, Spetzler–Martin grade 2, and have a low risk of rupture [46].

The mutational spectrum of HTT includes the ENG and activin receptor-like kinase type 1 (*ALK1*) gene, distinctive for the HHT type 1 and 2, respectively [47,48]. The decapentaplegic homolog 4 (SMAD4) and BMP gene were also identified in HHT patients [47,48,49,50]. 

HHT-related genes are related to the TGF-β pathway, which proved to be pivotal in regulating endothelial expansion, differentiation, and matrix remodeling [51,52]. HHT-mutations crosstalk even with the VEGF, PTEN, PI3K/AKT, and MAPK/ERK activity, which regulates the physiological mechanism of angiogenesis [53,54,55]. This evidence, also supported by studies on genetic mouse models, supports the hypothetical role of the aberrant pathways underlying the etiology and pathogenesis of hereditary bAVMs [56,57,58].

Moreover, the analyses revealed sporadic aberrations and single nucleotide polymorphisms predisposing to de novo cerebral vascular malformations [13,59,60]. The mechanism of inheritance of sporadic bAVMs is still not well elucidated. The main gene mutations, identified as genetic risk factors for bAVMs occurrence, involve the RAS-related pathways. 

Recent studies documented the prevalence of activating KRAS-related mutations in ECs of sporadic bAVMs [61,62,63]. The increased KRAS activity upregulates several downstream molecular pathways, such as PI3K/AKT/mTOR and MAPK/ERK. PI3K routes control angiogenic mechanisms, including VEGF, by regulating the proliferation, migration, and survival of ECs [64,65]. MEK/ERK1-2 promotes cell growth, progression, and genetic instability [66,67]. The high function of the PI3K/AKT pathway, and thus the VEGF signal, may control the BMP9, ENG, ALK1, and SMAD4 activities. These last interfere with the endothelial function and hemodynamic reaction, resulting in nidus remodeling and progression [68,69].

In 2022, Wang et al. recruited a cohort of 150 patients with bAVMs and performed whole-exome sequencing on peripheral blood DNA, to investigate the mutational spectrum [70]. Results revealed a strong correlation with the aberration of the RAS-RAF-MEK-ERK pathway, particularly the presence of the RASA1 mutation, an autosomal dominant disorder related to vasculogenesis [64].

These mutations within the endothelial genome are responsible for the steady bAVMs vascular reshaping, also in concurrence with the pulsatility of blood flow. The continuous wall shear stress alters the chromatin assembling, DNA methylome, and endothelial mechanotransduction, leading to bAVM development [71,72,73]. The endothelial dysfunction reflects an increased activation of the inflammatory cascade, proangiogenic mediators, matrix proteins, and growth factors triggering tube proliferation [74,75,76,77,78].

Among these vascular disorders, high flow shunt, dysfunction of vascular cells, inflammatory cell infiltration, and matrix remodeling may cause vessel wall weakness and predispose to bAVMs rupture. The molecular mechanisms underlying the risk of bAVMs breakage are recognizable in the mutations of angiogenesis pathways and inflammation signals.

On these assumptions, the analysis of the miRNAs is intended to deepen the genetic aspects which upstream regulate and support the molecular pathways involved in cerebral vascular malformations.

Our review retrieved four articles sustaining the theoretical role of miRNAs in the bAVMs pathogenesis. Genetic investigations were conducted on the ECs, VSMCs, and peripheral blood [29,30,31,32].

miRNA-18a was reported as downregulated on bAVM-ECs, influencing the activation of matrix reprocessing factors, Id-1, HIF-1α signals, and the VEGF pathway [29,30]. Restoration of the physiological miRNA-18a expression was showed to inhibit bECs anomalous proliferation, migration, and bAVM development [29,33]. 

In 2017, Huang and his group investigated the miRNAs’ influence on bAVM-VSMCs properties [31]. VSMCs are fundamental in supporting vascular structure, as they sustain the stiffness of vessels wall and resilience against blood flow. VSMCs contribute to the bAVMs’ progression by heading the arterialization of the venous drainages and managing the molecular interactions which promote vasculogenesis and inflammation [79]. miR-137 or miR-195* were found under-expressed in the bAVM-VSMCs, while the miRNA-related pathways were upregulated [31]. 

The increase of target regulatory networks, such as the VEGF, NFkB, PI3K/Akt, and MAPK signals, enhanced bAVM-VSMCs migration and tube formation. Additionally, miR-137 and miR-195* may affect the phenotypic switching of VSMCs, through cytokine and growth factors secretion, resulting in the inhibition of aberrant vascular remodeling. 

Further analysis of serum samples identified the levels of the top five bAVM-related miRNAs as differentially increased [32]. The high levels of miR-7-5p, miR-629-5p, miR-199a-5p, miR-200b-3p, and let-7b-5p triggered the abnormal angiogenesis, vascular rearrangement, and stimulation of VEGF, MMPs, and inflammatory mediators. 

The bioinformatics analysis we performed using artificial intelligence algorithms confirmed the correlation between miRNA-related target genes and the above-mentioned molecular mechanisms underlying bAVMs growth. The functional enrichment investigation revealed several intracellular signals allegedly involved. These included firstly the VEGF cascade, renowned for angiogenesis; HIF-1α, transcribed in reaction to hypoxia; PI3K/Akt, MAPK/ERK, and NFkB pathways implicated in cell survival; ECs proliferation; and inflammation.

In 2021, Florian and colleagues conducted a comprehensive review of the role of miRNAs in the pathogenesis and progression of bAVMs and cerebral cavernous malformations [23]. Their findings, following our results, reported the downregulation of miR-18a, miR-137, and miR-195* in bAVMs and high blood levels of miR-7-5p, miR-199a-5p, miR-200b-3p, and let-7b-3p. Two miRNAs were found to be upregulated in human cavernous malformations, namely the miR-27a and mmu-miR-3472a, while the miR-125a, miR-361-5p, miR-370-3p, miR-181a-2-3p, miR-95-3p, and let-7b-3p were found to be upregulated. 

Their conclusions strongly support the significance of miRNAs in the occurrence and hemodynamics of neurovascular malformations [23].

Apart from supporting the pathogenesis of vascular abnormalities, miRNAs should be interpreted as prognostic markers as well as innovative diagnostic tools. Identification of specific miRNAs within the bAVMs microenvironment allows for designing novel therapeutic strategies and also improving the existing practices.

### 4.1. Experimental Strategies and Future Perspectives for bAVMs

Therapeutic management algorithms for bAVMs are constantly improved by the design of hybrid surgical, endovascular, and radiotherapy protocols. The up-to-date guidelines refer to tailored grading systems that stratify the individual risk and allow the choice of the most appropriate treatment [80,81].

Advances in translational medicine and genetics allowed improvements of the therapeutic approaches for vascular malformations in bAVMs equal to those in the neurooncological field [82,83,84,85,86,87,88,89,90,91,92,93,94]. Novel pharmacological regimes consider the genetic factors behind bAVMs genesis and rearrangement and experiment-refined biological and molecular strategies. 

Based on the substantial implications of the VEGF pathway in the bAVMs vascular remodeling, it was the first candidate as a therapeutic target. In animal studies, bevacizumab, an anti-VEGF monoclonal antibody, was shown to reduce the progression of cerebral vascular malformations [95,96]. A phase I clinical trial, completed in 2020, tested the bevacizumab in bAVMs treatment. It reported efficacy in reducing the size of lesions and no side effects were reported (#NCT02314377).

Despite the administration of soluble VEGFR has exhibited antiangiogenetic properties in the AVMs mouse model, no further strategies were planned due to the risk of simultaneous inhibition of the proper cerebral vasculature [97,98].

A class of antibiotics, tetracyclines—especially doxycycline—, was tested as bAVM therapy [99,100]. Doxycycline was employed as monotherapy, or combined with minocycline, in three already completed phase I clinical trials, (https://clinicaltrials.gov (accessed on 16 July 2022) #NCT00783523, #NCT00783523, #NCT00243893). It demonstrated significant activity against vascular proliferation and tube formation with a good safety profile. Doxycycline also proved to inhibit MMP remodeling, reducing the risk of rupture [99]. 

The most recent phase II clinical trial exploits the anti-inflammatory and antiproliferative properties of lovastatin for bAVMs ECs and VSCMs (#NCT04297033). Results are still pending. Furthermore, the intravenous administration of naked miR-18a showed effective results. miR-18a controlled the ECs function via an easy intracellular diffusion, without a need for transfection reagents, resulting in the inhibition of tube development and vascular proliferation [22,29]. 

### 4.2. Emerging miRNA-Based Therapies

miRNA-based therapeutics can be feasible and effective noninvasive strategies and they are classified as mimics or inhibitors (antimiRs). Mimics are synthetic double-stranded molecules that directly link miRNAs restoring missing expressions, while the antagonists inhibit specific miRNA targets. 

Several miRNA-based drugs are under examination in phase I and II clinical trials, revealing potential efficacy for the treatment of tumors, hepatitis C, atherosclerosis, and kidney diseases.

In cancer therapy, miRNAs may control the tumor progression via the expression/repression of target genes aimed at regulation of the cell cycle, metabolism, apoptosis, and immunosuppression. The overexpression of miR-21 in tumors blocks the activity of oncosuppressor genes. The antimiR-21, tested in breast cancer, was demonstrated to inactivate the AKT and MAPK pathways, which are related to cancer cell proliferation, angiogenesis, and chemo-resistance [101,102]. The miR-34 is was found to be under-expressed in neuroblastoma, lung cancer, melanomas, and leukemias [103,104]. The oncosuppressive activity of miR-34, exploited via the p53 pathway, was employed as a mimic therapeutic agent and showed promising results [105,106].

The miR-122 has a fundamental role in liver cell metabolism and is involved in the assembly of the hepatitis C virus (HCV) [107,108]. antimiR-122 drugs were tested in clinical and preclinical models, via intravenous administration, showing dose-dependent inhibition of HCV replication [109,110,111]. Unfortunately, a severe side effect was reported, namely the quick reduction in plasma cholesterol levels [112,113].

Furthermore, the miR-33a/b inhibits the expression of the ABCA1, a cholesterol transporter in the liver cells [114]. Inhibitors of miR-33a/b proved to enhance the expression of ABCA1, resulting in the increase of plasma HDL level, an atheroprotective effect, and subsequent plaque regression [114,115].

Numerous microRNAs are involved in the progression of renal fibrosis, including the miR-2. miR-2 is an excellent candidate for therapy because it regulates the redox metabolic pathway and lipid metabolism by the peroxisome proliferator-activated receptor-a (Ppar-a) [116].

miRNA therapeutics are even usable in the treatment of neurovascular malformations, to prevent vascular remodeling and decrease aberrant angiogenesis.

In a clinical study, Marín-Ramos et al. tested the miR-18a, via intravenous and intranasal administration, as a noninvasive therapy for bAVMs. They reported the efficacy of miR-18a in boosting TSP-1 activity, inhibiting VEGF pathways and AVM-BEC dysfunction [30].

Despite the encouraging results of miRNA-based therapies, the poor bioavailability, restricted tissue permeability, payload instability, and choice of delivery systems are still the main concerns [117].

Brain administration is further complicated by the cross of the blood–brain barrier (BBB), through which only lipid-soluble small molecules of less than 400 Daltons can penetrate. 

Liposomes and nanoparticles were tested as miRNA vectors, but the results are still forthcoming [117,118]. Another developing strategy was the transient disruption of the BBB via chemical agents or alcohols [119]. In the study conducted by Marín-Ramos, they combined the miR-18a with the NEO100, a perillyl alcohol, aiming to facilitate the miR-18a delivery through the BBB [30].

Although the miRNA-based therapies were not yet approved by the FDA, advances in genetic engineering and translational medicine may lead to identifying new valid miRNA therapeutics to be translated from the bench to the bedside.

### 4.3. Limitations

The present study has several potential limitations: first, the limited number of studies involved; second, the scarcity of the genetic data reported; third, the intrinsic biases of artificial intelligence algorithms. 

Additional limitations lie in the relative rarity of cerebral vascular malformations and the lack of certain knowledge about mechanisms underlying bAVMs pathogenesis. Therefore, results should be interpreted with caution.

## 5. Conclusions

bAVMs are high-flow vascular disorders that undergo constant remodeling under multifarious genetic and hemodynamic stimuli.

miRNAs are important for genetic transcription regulation and modulation of intracellular pathways implicated in the vasculogenesis and proliferation signals. 

The downregulation of miRNA-18a on the ECs may justify the lack of TSP-1 transcription and the overexpression of Id-1, proangiogenic factors, PAI-1, BMP4, HIF-1α, and MMPs, which leads to vessels rearrangement and bAVM growth. 

On the VSMCs, the downregulation of miR-137 and miR-195* should affect the boost of VEGF, PI3K/Akt, MAPK/ERK, and NFkB pathways. These last induce the VSMCs phenotypic switching, vessel hypertrophy, and activation of the inflammatory cascade. 

The increased levels of miR-7-5p, miR-629-5p, miR-199a-5p, miR-200b-3p, and let-7b-5p in the peripheral blood samples triggered remodeling of extracellular matrix, aberrant angiogenesis, and nidus formation. 

miRNA identification may act as a pivot for designing novel diagnostic and prognostic implements, as well as innovative drug mimetics of the miRNAs. Further advances are needed to implement our knowledge about bAVMs’ pathophysiology and refine their treatment regimens.

## Figures and Tables

**Table 1:** Dysregulated microRNAs and direct target genes in brain arteriovenous malformations.

miRNA	Site	Status	Level of Gene Expression		Author, Year	Country
			High	Low		
miR-18a	BECs	Downregulated	TSP-1, VEGF-A	Id-1, VEGF-D	Ferreira et al., 2014 [29]	USA
			TSP-1	VEGF, PAI-1, BMP4, HIF-1α MMP2, MMP9, ADAM10	Marín-Ramos et al., 2020 [30]	USA
miR-137	VSMCs	Downregulated	NA	VEGF, PI3K/Akt, MAPK/ERK, P38, NFkB	Huang et al., 2017 [31]	China
miR-195*						
miR-7-5p	PB	Over-expressed	VEGF	NA	Chen et al., 2018 [32]	China
miR-629-5p						
miR-199a-5p						
miR-200b-3p						
let-7b-5p			NA			

ADAM10: ADAM metallopeptidase domain 10; Akt: Ak strain transforming; BECs: brain endothelial cells; BMP4: bone morphogenetic protein 4; ERK: extracellular signal-regulated kinase; HIF-1α: hypoxia-inducible factor 1α; Id-1: inhibitor of DNA binding 1; MAPK: mitogen-activated protein kinase; miRNA: microRNA; MMP: matrix metalloproteinase; NA: not available; NFkB: nuclear factor kappa-light-chain-enhancer of activated B cells; PAI-1: plasminogen activator inhibitor-1; PB: peripheral blood; PI3K: phosphatidylinositol 3-kinase; TSP-1: thrombospondin-1; VEGF: vascular endothelial growth factor; VSMCs: vascular smooth muscle cell.

## Data Availability

All data presented in the research are included in this article.
